# Diffusion of Molecular Diagnostic Lung Cancer Tests: A Survey of German Oncologists

**DOI:** 10.3390/jpm4010102

**Published:** 2014-03-21

**Authors:** Julius Alexander Steffen

**Affiliations:** ZS Associates International, Inc., Neue Mainzer Straße 28, 60311 Frankfurt, Germany; E-Mail: julius.steffen@zsassociates.com; Tel.: +49-151-1661-4680

**Keywords:** diffusion of innovations, diagnostic tests, lung cancer

## Abstract

This study was aimed at examining the diffusion of diagnostic lung cancer tests in Germany. It was motivated by the high potential of detecting and targeting oncogenic drivers. Recognizing that the diffusion of diagnostic tests is a *conditio sine qua non* for the success of personalized lung cancer therapies, this study analyzed the diffusion of epidermal growth factor receptor (EGFR) and anaplastic lymphoma kinase (ALK) tests in Germany. Qualitative and quantitative research strategies were combined in a mixed-method design. A literature review and subsequent Key Opinion Leader interviews identified a set of qualitative factors driving the diffusion process, which were then translated into an online survey. The survey was conducted among a sample of 961 oncologists (11.34% response rate). The responses were analyzed in a multiple linear regression which identified six statistically significant factors driving the diffusion of molecular diagnostic lung cancer tests: reimbursement, attitude towards R&D, information self-assessment, perceived attitudes of colleagues, age and test-pathway strategies. Besides the important role of adequate reimbursement and relevant guidelines, the results of this study suggest that an increasing usage of test-pathway strategies, especially in an office-based setting, can increase the diffusion of molecular diagnostic lung cancer tests in the future.

## 1. Introduction

Among all cancer types, lung cancer is the most mortal cancer worldwide and has experienced the least improvements in terms of patients’ survival rates between 1977 and 2006 [1,2]. High mortality rates combined with relatively small improvements over time imply that the need for therapeutic progress is especially high in this disease field. Hence, personalized medicine as an innovative treatment approach appears particularly relevant to lung cancer patients.

The benefits of molecular diagnostic lung cancer tests have been shown in several studies [3,4,5]. A U.S. multi-center study presented at the 2013 American Society of Clinical Oncology (ASCO) Annual Meeting showed that detecting and targeting oncogenic drivers increases lung cancer patients’ survival rates [6]. This suggests that the diffusion of this treatment approach is desirable from a medical and social viewpoint. Current treatments targeting oncogenic drivers such as epidermal growth factor receptor (EGFR) and anaplastic lymphoma kinase (ALK) are approved as companion diagnostics, *i.e.*, their prescription must be based on the result of a corresponding diagnostic test. This makes diagnostic testing a *conditio sine qua non* and implies a dual diffusion process: the diffusion of targeted lung therapies directly correlates with the diffusion of corresponding diagnostic tests. 

Existing market research reports have shown that the diffusion of existing diagnostic lung cancer tests—which according to the molecular testing guidelines of the International Association for the Study of Lung Cancer are mainly EGFR and ALK—is still low ([7], Pfizer Inc., New York, NY, USA, data on file [8]). Looking for reasons for the slow diffusion of molecular diagnostic lung cancer tests in existing literature has not led to any results at the initiation of this study. Against that background, this study sought to identify and statistically assess factors which drive the diffusion of diagnostic lung cancer tests.

## 2. Methodology

### 2.1. Research Design

The study was divided into two major parts requiring different research strategies. The first part was focused on the inductive identification of factors driving the diffusion process. This required qualitative strategies of inquiry, such as a review of relevant literature and expert interviews. The subsequent part sought to assess the statistical significance and strength of association between each identified factor and the diffusion process. The data required for the statistical analysis was produced in survey research. In this manner, the study integrated qualitative and quantitative data—an approach which is generally defined as mixed method research [9].

### 2.2. Population and Sampling Approach

The study population consisted of German oncologists. The German healthcare system was chosen for two major reasons: firstly, Germany is a particular interesting case, since compared to other European healthcare systems it has high expenditures on health *per capita* (In 2011, Germany spent $4495 on healthcare *per capita*, whereas France, Spain, Italy and the UK spent $4118, $3072, $3012 and $3405 respectively) [10], yet, the usage of diagnostic lung cancer tests as captured in past market research reports has been relatively low compared to Italy and France, for example (Pfizer Inc., data on file [8]). Secondly, Germany plays a central role in the diffusion of a medical innovation within Europe because of its role as a reference price country.

Pathologists were not included in the study population, since they do not directly impact the diffusion process. Although they conduct the actual diagnostic test, the German healthcare system dictates that the pathologist is only allowed to test a patient if this test has been ordered by an oncologist. According to the German Federal Health Monitoring, 3422 practitioners were holding an additional postgraduate education in “tumor therapy with drugs” in 2012. 82.8% were male and 7.2% were female. 55.3% were office-based, 43.4% were hospital-based, and 1.3% was working in other institutions and corporate bodies. 40.1% were between 40 and 50 years old while 32.1% were between 50 and 60 years old [11].

The population was stratified according to the work setting: hospital- *vs.* office-based oncologists. Office-based oncologists were accessed through the German professional association of office-based oncologists “*Bund für Niedergelassene Onkologen und Hämatologen*” (BNHO). The BNHO has more than 575 members, treating approximately 300,000 patients in over 360 offices every year [12]. The advantage of using the BNHO for sampling purposes is that although the sample remains non-probable as not all oncologists are registered at the BNHO and not all of them have an email address, it is representative in terms of geography. 

Hospital-based oncologists were identified by visiting the websites of the hospitals in all sixteen states of Germany. The list of hospitals was derived from the German hospital directory [13].

### 2.3. Research Implementation and Validation

The qualitative part of the study used two distinct data collection strategies: a literature review and a subsequent Key Opinion Leader (KOL) interview. The literature review was performed in a narrative way including relevant diffusion studies from three different fields: (1) diffusion of innovations; (2) diffusion of medical innovations; and (3) diffusion of diagnostic tests. As the review addressed medical and economic issues, a diverse range of databases were searched: EBSCO and EconLit covered the economic part of the review, whereas PubMed, MEDLINE and Ovid were used for medical studies.

The diffusion of diagnostic tests in lung cancer represented a literature gap when this study was initiated as there were no studies examining the diffusion process of molecular diagnostic lung cancer tests. Hence, the factors identified in existing literature were related but not specific to the case of lung cancer. This is why semi-structured KOL interviews were conducted to validate the identified factors to the concrete case of lung cancer. 

After identifying and validating general factors channeling the diffusion of diagnostic tests in lung cancer, the statistical relationship between each individual factor and the overall diffusion process was examined in the quantitative research part. The identified factors were translated into a web-based survey which was piloted with two final-year medical students in order to anticipate problems and avoid wastage. The results from the pilot were incorporated into the final survey design. To decrease non-response, the survey participation was incentivized.

The survey data was analyzed in a multiple linear regression. In addition to that, Pearson’s linear correlations were calculated in order to analyze the relationships among the independent variables, which supplemented the results of the multiple linear regression. The results of the quantitative analysis were validated by comparing them to existing studies examining the diffusion of molecular diagnostic tests in other disease fields.

## 3. Results

### 3.1. Qualitative Research Findings

The literature review showed that the diffusion of innovations can be seen as the sum of the individual adoption decisions of the members of a social system. For this study, oncologists have been identified as those individuals who have to make the adoption decision. Hence, the diffusion of molecular diagnostic lung cancer tests is the sum of the individual decisions of oncologists about whether or not to use testing. While the outcome of this decision largely depends on the characteristics and perceptions of the individual oncologist, it is equally influenced by other parties such as pathologists, payers, colleagues, and others. Understanding which factors influence an oncologist in his or her adoption decision was therefore critical in understanding the diffusion process.

Table 1 summarizes the factors which could be identified in the literature review and validated in the KOL interview.

**Table 1:** Factors driving the diffusion of diagnostic lung cancer tests in Germany.

Factor	Description
Work setting	The Key Opinion Leader (KOL) interviews suggested that in Germany the work setting has implications on reimbursement: while the costs of diagnostic tests ordered by office-based oncologists are generally fully reimbursed, the costs of tests ordered by hospital-based oncologists are reimbursed by a lump sum as defined by “diagnosis related groups”. Practice variation studies additionally show that the work setting influences physicians’ work patterns [14].
Trial participations and attitude towards R&D	The KOL interviews pointed out that trial participations are positively correlated with the oncologist’s knowledge and general attitude towards innovations. In this context, KOLs see a cultural issue in the specific case of Germany: whereas in some countries such as the U.S. innovations are generally positively perceived, the German culture does not always appreciate innovative outcomes of medical research and development to the same extent.
Infrastructure	A lack of infrastructure can slow down the diffusion of innovations [15]. The KOL interviews highlighted that tests may not always be locally available.
Test-pathway strategies	Test-pathway strategies can potentially standardize and simplify the collaboration between oncologists and pathologists by defining the type and sequence of tests which are conducted for all patients. The importance of interdisciplinary collaboration is confirmed in the KOL interviews.
Cost reimbursement	A variety of studies discussing the diffusion of molecular diagnostic tests point out that a lack of reimbursement can significantly slow down the diffusion process [16,17,18]. This is confirmed in KOL interviews, highlighting reimbursement as the most relevant factor.
Information and knowledge	Existing diffusion studies suggest that a lack of knowledge and information can slow down the diffusion process [7,19]. This confirmed in the KOL interviews emphasizing that there is a lack of knowledge concerning targeted lung cancer therapies and respective diagnostic tests among German oncologists.
Complexity and compatibility	Studies on the adoption of molecular diagnostics show that novel diagnostic technologies can complicate patient management and may lead to an information overload [20]. Additionally, the diffusion is faster for technologies which are perceived as ordinary rather than revolutionary and fit in with existing procedures and beliefs.
Value of diagnostics	The perceived value of an innovation is an integral part of general diffusion research. An innovation must add value in order to be adopted and the added value must be visible to the adopter [21,22].
Attitude of colleagues	This variable is related to the role of the oncologists’ social context emphasized in existing studies suggesting that physicians who work together as colleagues gradually adapt to each other and thus become alike [14,23].
Consensus among colleagues	This variable is related to the role of the social context [9,14,24] as well as to the factors of evidence and uncertainty [21,25]: an individual who associates a personal risk with an innovation is less likely to adopt it. The risk associated with an innovation results from the uncertainty surrounding its value. A perceived consensus among colleagues reduces uncertainty and the risk associated with an innovation.
Strength of evidence	Diagnostics specific diffusion studies [7,16,17] have shown that scientific evidence serves as a major source of value judgment. Yet, clear evidence often does not emerge until an innovation has been introduced and experimented in practice, which can lead to a slow diffusion process as uncertainty persists. This has been confirmed in the KOL interviews.
Reliance on biopharmaceutical industry	The influence of marketing and communication activities of biopharmaceutical companies has been identified in the context of diffusion of medical innovation [26,27] and is assumed to positively correlate with the diffusion process.

### 3.2. Quantitative Research Findings: Descriptive Analysis

#### 3.2.1. Survey Sample

The survey was sent out to 961 oncologists: 582 office-based and 379 hospital-based. 85 surveys could not be delivered and 109 surveys were completed, which equates to a response rate of 11.34%. The mean age of respondents was 49.45 years (Table 2). 89% of respondents were male, 11% female, and 61% worked in a hospital whereas 39% were office-based. While the distributions of age and gender were similar to the actual population, hospital-based oncologists were over-represented in the sample (61% of the sample *vs.* 43.3% of the actual population). However, as the survey controlled for the setting in which the respondents worked, the over-representation of hospital based oncologists did not cause any bias.

**Table 2:** Descriptive results.

Variable	Valid responses	Minimum	Maximum	Mean
Diagnostics usage rate	109	0	100	64.14
Age	107	30	75	49.45
Gender	109	0	1	0.89
Work setting	109	0	1	0.61
Trial participations	107	0	432	19.85
Infrastructure	109	0	1	0.93
Test pathway strategies	109	0	1	0.53
Cost reimbursement	109	1	3	2.31
Information and knowledge	109	4	10	8.61
Perceived value of diagnostics	109	2	10	8.08
Perceived attitude of colleagues	109	3	10	7.28
Perceived consensus among colleagues	109	2	10	7.2
Perceived strength of evidence	109	2	10	7.73
Attitude towards R&D	109	2	10	7.35
Reliance on biopharmaceutical industry	109	1	10	5.72

#### 3.2.2. Current Testing Practices

**Figure 1:**
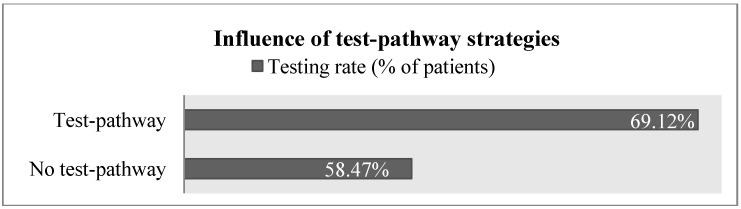
Interplay between test-pathway strategies and testing rates.

Testing rates ranged from 0% to 100% of lung cancer patients. The mean testing rate was 64.14% of patients, with a standard deviation of 27.76%. In terms of infrastructure and test availability, 93% of respondents indicated that both ALK and EGFR tests are locally available. All respondents collaborated with a pathologist but only 53% were using test-pathway strategies. This is an important finding since on average, oncologists using a test pathway order more diagnostic tests than oncologists who discuss the case of each patient individually (Figure 1). The mean testing rate of respondents using a test-pathway was 69.12% of patients while the mean rate of those not using a test-pathway was 58.47%.

#### 3.2.3. Reimbursement

**Figure 2:**
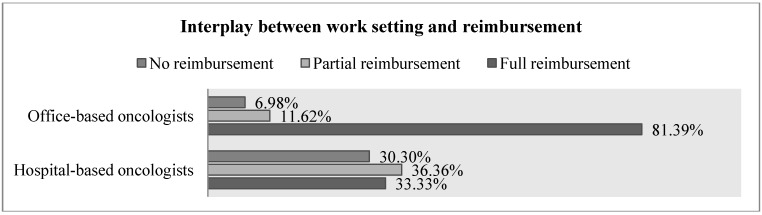
Interplay between work setting and reimbursement.

Concerning the degree to which the costs of conducting a diagnostic lung cancer test are reimbursed, 52.29% of respondents indicated full reimbursement, whereas 26.61% indicated partial reimbursement and 21.10% indicated no reimbursement at all. This is an interesting finding since the qualitative research only differentiated between full and partial reimbursement, *i.e.*, it suggested at least some degree of reimbursement. Office-based oncologists indicated higher levels of reimbursement than hospital-based oncologists (Figure 2): full reimbursement was claimed by 81.39% of office-based oncologists and only 33.33% of hospital-based oncologists.

#### 3.2.4. Perception of Lung Cancer Diagnostics

On average, respondents saw molecular diagnostic lung cancer tests as a technology which adds value and which is well perceived among colleagues: the perceived value added by diagnostic tests was relatively high with a mean value of 8.08 out of 10 while the perceived attitude of colleagues had a mean value of 7.28. Also the perceived consensus among colleagues and the perceived strength of evidence were relatively strong with mean values of 7.20 and 7.73, respectively. On a scale from one to ten, the mean value of information self-assessment was 8.61, *i.e.*, on average, the respondents felt well informed about diagnostic tests in lung cancer. Concerning the source from where oncologists receive their information about diagnostic lung cancer tests, the importance of the biopharmaceutical industry as an information provider received a mean value of 5.72 and ranged from one to ten.

### 3.3. Quantitative Research Findings: Multiple Linear Regression

The multiple linear regression was performed with different specifications and the results were robust to all specifications (Table 3). Excluding statistically insignificant variables leaves six significant variables. These are: reimbursement (*p* = 0.00003), attitude towards R&D (*p* = 0.0003), information self-assessment (*p* = 0.005), perceived attitudes of colleagues (*p* = 0.007), age (*p* = 0.029) and test-pathway strategies (*p* = 0.033).

The standardized coefficient β allows for comparisons between the type and strength of influences the different variables exert on the testing rate. Except for the oncologists’ age, all significant variables were positively correlated with the testing rate. Reimbursement and attitude towards R&D have the largest coefficients, implying that the mean testing rate of lung cancer patients changes most strongly if the degree to which costs are reimbursed or the attitude of oncologists towards medical R&D change, holding the other variables constant: For instance, when reimbursement is increased from none to partial or from partial to full, the testing rate increases at an average rate of 12.18%.

Some variables which were not statistically significant in the regression did have statistically significant correlations according to Pearson with information and knowledge, reimbursement, and test pathway strategies. The perceived strength of evidence had a significant positive correlation with information and knowledge which, in turn, was a significant variable in the regression. Also, the perceived value of diagnostic tests significantly correlated with the level of information and knowledge, indicating that the more information an oncologist has, the more likely is he or she to have a positive perception of the value of diagnostic lung cancer tests. The work setting significantly correlated with the level of reimbursement and with the usage of test-pathway strategies, indicating that test-pathways are more likely used in hospitals than in offices.

**Table 3:** Quantitative results: Exponentiated coefficients; cursive standard coefficients. * *p* < 0.1; ** *p* < 0.05; *** *p* < 0.01.

Variable	Model 1	Model 2	Model 3
	R^2^ = 0.417	R^2^ = 0.395	R^2^ = 0.393
Reimbursement	10.526 ***	11.467 ***	12.180 ***
	*0.305*	*0.329*	0.35
Attitude towards R&D	3.969 ***	4.534 ***	4.515 ***
	*0.265*	*0.302*	*0.301*
Information and knowledge	4.391 **	4.605 **	4.605 ***
	*0.226*	*0.241*	*0.241*
perceived attitude of colleagues	3.245 *	3.830 *	3.945 ***
	*0.188*	*0.224*	*0.231*
Age	−0.624 **	−0.679 **	−0.638 **
	−*0.171*	−*0.176*	−*0.178*
Test pathway strategies	9.641 **	10.161 **	9.644 **
	*0.175*	*0.184*	*0.174*
Work setting	−4.582	−3.056	
	−*0.082*	−*0.054*	
Gender	−3.889	−1.083	
	−*0.045*	−*0.012*	
Reliance on biopharmaceutical industry	1.384		
	*0.108*		
Infrastructure	7.159		
	*0.069*		
Perceived consensus among colleagues	−0.431		
	−*0.033*		
Trial participations	−0.019		
	−*0.03*		
Perceived value of diagnostics	1.159		
	*0.086*		
Perceived strength of evidence	−0.37		
	−*0.023*		
Observations	109	109	109

## 4. Discussion

### 4.1. Major Findings and Implications for Clinical Practice

The results of this study show that there are large differences concerning the extent to which oncologists use diagnostic lung cancer tests (*rang*e: 100%), indicating that oncologist differ in terms of their innovativeness. Yet, on average, more than half of lung cancer patients are being tested in Germany, which matches with the author’s expectations based on existing market research. Oncologists consider diagnostic tests an innovation which adds value to the field of lung cancer as expressed by the high average scores of perceived value added and perceived attitudes of colleagues. The statistically significant role of the social context implies that the positive perception of diagnostic testing among oncologists can be expected to further drive its diffusion in the future, as less innovative oncologists will be encouraged to catch up by their peers. This complies with the findings of practice variation studies [28].

Apart from the significant role of the social context in the diffusion process, there are three major findings with relevant implications for clinical practice:

**1. Increasing usage of test-pathway strategies can accelerate the diffusion process**

The statistical significance of test-pathway strategies highlights the importance of interdisciplinary collaboration between oncology and pathology for the diffusion of molecular diagnostic lung cancer tests. The descriptive analysis of the survey responses showed that only 53% of respondents were using test-pathway strategies and that those respondents using test pathways had a higher average testing rates. This suggests that by increasing the usage of test-pathway strategies in clinical practice, the diffusion of molecular diagnostic lung cancer tests can be accelerated. Pearson’s correlations showed that the test-pathway strategies were more likely to be used in hospitals. Hence, the potential for increasing the usage of test pathway strategies is particularly high in the setting of office-based oncologists.

**2. Reimbursement plays a crucial role in the diffusion process, and existing schemes may need to be revised**

The results of this study show that reimbursement is one of the most critical factors in the diffusion process of diagnostic lung cancer tests. This meets the author’s expectations as the role of reimbursement had been a major finding of the qualitative research. This is a result with major practical implications, showing that payers play an essential role in the diffusion of diagnostic tests in Germany.

Surprisingly, one fifth of respondents indicated no reimbursement of molecular diagnostic lung cancer tests. This was unexpected because the literature review validated by the KOL interview only differentiated between partial and full reimbursement. This may be due to a lack of knowledge among practitioners about the reimbursement of diagnostic tests, or due to a lack of adequate reimbursement schemes which translate into clinical practice.

From the payers’ perspective, a required necessity for granting reimbursement is clear evidence of the clinical utility of diagnostic tests [16,17,29]. However, if not reimbursed, the diffusion of a test in clinical practice and consequently the generation of clinical data are hardly possible. A possible solution to this problem is provided by innovative approaches to reimbursement, such as risk-sharing schemes between payers and biopharmaceutical companies: a newly developed diagnostic test would initially be only partly reimbursed and receive full coverage once the clinical value has been demonstrated [30].

**3. Clinical guidelines reduce uncertainty, increase self-perceived knowledge and thereby drive the diffusion process**

A surprising survey result was that on average, the respondents felt well informed about diagnostic testing in lung cancer. This contradicts the results of most diffusion studies in the field of diagnostic testing, which have repeatedly outlined the lack of information and knowledge as a major barrier to diffusion: in a recent study examining the diffusion of diagnostic tests by surveying 10,303 U.S. physicians, only 12.9% felt adequately informed [19]. Although the sample of that study was more robust, only 1.3% of it comprised oncologists. Given that diagnostic testing and personalized medicine have been primarily introduced to the field of oncology, it seems reasonable that only surveying oncologists leads to higher self-reported levels of information. This is confirmed by a similar survey conducted in Canada, showing that the self-reported level of information about diagnostic testing is four times higher for oncologists as compared to family physicians [31]. Yet, also in that study, only roughly 40% of oncologists felt adequately informed.

The reason for the high level of self-reported information is likely to be the recent introduction of relevant guidelines [7]. Existing studies suggest that (1) the vast majority of physicians base treatment decisions on clinical guidelines; and (2) a lack of clinical guidelines leads to low levels of information and knowledge [18,30], which, in turn, slows down the diffusion process [31,32]. A recent study states that “there is a consensus that molecular testing of the lung carcinoma should be the standard of care in the clinical management of patients with lung carcinoma” [33]. This is confirmed in this study as the perceived consensus and strength of evidence are given relatively high average values. Although these variables are statistically insignificant in the regression, they correlate with the level of information and knowledge at a statistically significant level. In that sense, the results of this study do comply with other surveys: the introduction of clinical guidelines seems to have increased the perceived strength of evidence and consensus, which in turn have increased the level of self-reported information. This shows that clinical guidelines play an important role in the diffusion of molecular diagnostic tests.

### 4.2. Limitations, Generalizability and Future Research

The results of this study answer the research question by identifying those factors which drive the diffusion of diagnostic tests in lung cancer at a statistically significant level. The significant variables, however, explain only 39.3% of the statistical variance observed in the testing rates. This implies that there are other relevant factors which have not been covered in the survey of this study. Likely, it would have been beneficial to the survey design if more expert interviews had been conducted to explore the research question in more qualitative detail. Hence, the identification and statistical evaluation of additional factors can be examined in future research.

As with any type of survey research, this study faced response and non-response bias. Oncologists who responded to the survey are likely to be interested in molecular lung cancer diagnostics and the overall concept of personalized medicine than those who did not respond. As the survey does not specifically control for the respondents’ interest in the topic, this may be reflected in the results. It is likely, for example, that a higher interest in the topic correlates with greater knowledge. Hence, in the descriptive analysis of the survey responses, the average value reported for information and knowledge may be higher than in reality.

The survey of this study specifically examined the diffusion of EGFR and ALK tests as the two main diagnostic lung cancer tests according to the relevant guidelines [7]. Especially emerging parallel testing approaches, multiplex testing and genome sequencing will most likely face a different diffusion process. The infrastructure variable, which has not been significant in this study, may then play a more important role, whereas test-pathway strategies will become less relevant. 

Lastly, the generalizability of the results is limited to Germany. It would be interesting to conduct a similar study in another country in order to compare the results and assess the importance of country-specific factors. It would be particularly insightful to analyze a country with a healthcare system different to the German system. France, for instance, has been reporting higher diffusion rates of diagnostic tests in lung cancer (Pfizer Inc., data on file [8]). It would be interesting to see if this has cultural reasons or is due to the more centralized structures of the French healthcare system.

## 5. Conclusions

This study fills in a current literature gap by examining the diffusion of molecular diagnostic lung cancer tests. Understanding the diffusion process is mandatory to unlocking the value of treatment concepts involving the identification and targeting of oncogenic drivers and, thereby, reaching the required therapeutic improvements in lung cancer. The factors identified as statistically significant highlight the importance of adequate reimbursement, sufficient information, involvement of practitioners into R&D, as well as standardized collaborations between oncology and pathology. This has significant implications for practitioners, policy makers and the biopharmaceutical industry. Yet, there is room for further research identifying and statistically evaluating factors driving the diffusion of diagnostic tests in lung cancer. Hence, this study is making a start which subsequent studies can build on by analyzing the diffusion process from various perspectives and in different national healthcare systems.
